# Starch–Citric Acid Adhesive: Preparation and Performance Study Catalyzed by p-Toluenesulfonic Acid

**DOI:** 10.3390/polym17233224

**Published:** 2025-12-03

**Authors:** Jiankun Liang, De Li, Zhongyou Luo, Yuqi Yang, Tong Meng, Chuchu Chen, Huali Li, Ningyuan Zuo, Qiuli Li, Hui Yang, Zhigang Wu

**Affiliations:** 1 College of Civil Engineering, Kaili University, Kaili 556011, China; dushimensheng@126.com; 2 School of Chemistry and Material Engineering, Zhejiang A&F University, Hangzhou 311300, China; lide@stu.zafu.edu.cn; 3 Yueyang Forestry Bureau, Yueyang 414000, China; luo15286446047@163.com; 4 College of Forestry, Guizhou University, Guiyang 550025, China; 15180721265@163.com (Y.Y.); 18188101139@163.com (T.M.); 17384255170@163.com (C.C.); 17385535230@163.com (H.L.); 19985000770@163.com (N.Z.); 13037863887@163.com (Q.L.)

**Keywords:** starch, citric acid, p-Toluenesulfonic acid, bio-based adhesive, plywood, bonding performance

## Abstract

This study investigates the application effects of p-toluenesulfonic acid (p-TsOH) as an efficient catalyst in the esterification reaction of starch–citric acid adhesives, aiming to successfully prepare plywood with good water resistance through lower hot-pressing temperatures. By precisely controlling the addition ratio of pTSA (0–10%), the multifaceted impacts on the adhesive’s curing behavior, bonding strength, water resistance, thermal stability, and microstructure were analyzed. The results demonstrate that pTSA substantially catalyzes the esterification crosslinking reaction between starch and citric acid. Differential scanning calorimetry (DSC) analysis reveals a significant reduction in the reaction peak temperature from 197.7 °C to 154.3 °C, which effectively lowers the hot-pressing temperature and provides more energy-efficient processing conditions for plywood production. When pTSA addition is within the range of 6–8%, the adhesive exhibits superior bonding performance and water resistance. Moreover, thermal stability is significantly enhanced and the microstructure becomes denser, collectively improving the overall performance of the plywood. This study not only provides a solid theoretical basis for the development of high-performance, environmentally friendly, starch-based wood adhesives but also offers strong technical support for the practical application of related technologies expected to promote the green and sustainable development of the wood adhesive industry.

## 1. Introduction

Wood adhesives play a vital role in the wood panel industry. Against the backdrop of global “dual carbon” goals and increasingly stringent environmental regulations, the currently dominant formaldehyde-based resins (such as urea–formaldehyde resin adhesive and phenol–formaldehyde resin adhesive) have excellent bonding properties. However, their raw materials are derived from non-renewable petroleum resources, and they release formaldehyde and other volatile organic compounds during preparation and use, posing a serious threat to human health and the environment [1,2,3,4]. In today’s context of environmental protection and sustainable development, the development and promotion of bio-based adhesives that are both environmentally friendly and renewable, and whose performance can match that of traditional resins, have become a focal issue of common concern in both academia and the industry and are an inevitable trend of the times [5,6,7,8,9].

In recent years, an increasing number of researchers have been committed to developing wood adhesives based on high-molecular-weight biomass raw materials (such as proteins, tannins, lignin, and carbohydrates). Among them, carbohydrate-based raw materials, such as starch, cellulose, and chitosan, have shown great potential for adhesive applications due to their unique chemical structures and rich functional groups [10,11,12,13,14,15,16,17]. Starch, as a widely available, inexpensive, and biodegradable polysaccharide natural polymer, is considered one of the ideal alternatives to traditional petroleum-based adhesives [18,19,20,21]. However, natural starch adhesives face many challenges in practical applications, such as insufficient bonding strength, poor water resistance, and high hot-pressing temperatures. These disadvantages seriously limit their widespread application [22]. Thus, overcoming the performance limitations of starch through efficient physical or chemical modification methods has emerged as a crucial step in advancing its industrial applications. To address the inherent shortcomings of natural starch, researchers have vigorously pursued a range of modification strategies, such as oxidation, crosslinking, and esterification. These approaches aim to substantially enhance the overall properties of starch adhesives, thereby broadening their potential applications in the adhesive industry [23,24,25].

Citric acid (CA), with three carboxyl groups and one hydroxyl group in its molecular structure, can undergo esterification with the hydroxyl groups in starch to form a stable three-dimensional crosslinked structure. The formation of this structure can significantly enhance the water resistance and bonding strength of starch adhesives, making it a bio-based adhesive with great development potential [20]. However, the esterification reaction between starch and citric acid is relatively slow. In the process of preparing wood adhesives, a high hot-pressing temperature is often required to promote the reaction [24]. In traditional esterification reactions, inorganic strong acids (such as hydrochloric acid and sulfuric acid) are commonly used as catalysts. However, when these inorganic acids are discarded after use, their slow degradation rate can cause serious environmental damage. Given this, the development of an efficient, mild, selective, and environmentally friendly catalytic system has become the key to the commercial application of high-performance starch–citric acid adhesives.

p-Toluenesulfonic acid (pTSA), as a strong organic acid, is unique in that it does not introduce oxidative factors that may disrupt the reaction, compared with traditional strong acids such as sulfuric and hydrochloric acids. This not only ensures the purity of the reaction but also significantly improves catalytic efficiency while minimizing the formation of by-products [26]. In recent years, pTSA has been successfully applied in many fields, such as biomass pretreatment and cellulose esterification, and its efficiency and selectivity have been widely recognized [27,28]. However, despite this, there are still many key issues that need to be resolved in the systematic application of pTSA to catalyze the crosslinking esterification reaction between starch and citric acid, specifically for the preparation of wood adhesives. At present, research on process optimization is still relatively insufficient, especially for the quantitative analysis of the impact of pTSA on the final bonding properties (such as dry strength, water resistance, and durability), which still needs in-depth and systematic research.

Therefore, this paper systematically studies the preparation process, curing behavior, bonding properties, and water resistance of starch–citric acid adhesives catalyzed by pTSA. This study aims to innovatively use pTSA as an efficient and selective catalyst to catalyze the esterification crosslinking reaction between corn starch and citric acid, thereby preparing a high-performance, environmentally friendly, starch-based adhesive for wood. The innovative aspects of this study are highlighted in several key points: first, the use of pTSA as a catalyst represents a novel approach to accelerate the esterification crosslinking reaction between starch and citric acid, significantly reducing the hot-pressing temperature and energy consumption. Second, the study provides a comprehensive analysis of the impact of pTSA on the bonding properties and water resistance of the adhesive, filling the gap in the current literature. Finally, the developed adhesive offers a sustainable and environmentally friendly alternative to traditional formaldehyde-based adhesives, aligning with the global trend towards green chemistry and sustainable development.

## 2. Materials and Methods

### 2.1. Materials

Corn starch and citric acid were sourced from Shanghai McLin Biochemical Technology Co., Ltd. (Shanghai, China). p-Toluenesulfonic acid was provided by Tianjin Huasheng Chemical Reagent Co., Ltd. (Tianjin, China). Poplar veneer (*Populus* spp., moisture content 8–10%), with dimensions of 400 mm × 400 mm and a thickness of 1.5 mm, was purchased from Shuyang, Jiangsu Province.

### 2.2. Preparation of Starch–Citric Acid Adhesive

Corn starch was mixed with citric acid in a mass ratio of 1:1 and dissolved in deionized water to prepare a 70% solution. The solution was then reacted in an oil bath at 120 °C for 1 h. After the reaction solution cooled to room temperature, pTSA was added at mass fractions of 0%, 2%, 4%, 6%, 8%, and 10%, respectively, and the mixture was stirred continuously for 10 min to ensure uniform mixing.

### 2.3. Plywood Preparation and Performance Testing

Three-layer poplar plywood was fabricated in the laboratory, with the adhesive applied at a rate of 160 g/m^2^ on one side of the poplar veneer. After assembly, the plywood was first subjected to a 10-min cold pressing, followed by hot pressing using a hot-press machine (model ST-15YP, purchased from Kunshan Lugong Precision Instrument Co., Ltd., Xiamen, China). The hot-pressing parameters were as follows: hot-pressing temperature was set at 150 °C, the unit hot-pressing pressure was 1.0 MPa, and the hot-pressing time was 1.2 min/mm. After hot pressing, the bonding strength of the three-layer poplar plywood was tested in accordance with the national standard GB/T 17657-2022 [29]. The final strength is the average of the 12 samples.

### 2.4. Residue Rate Test of Cured Products

Adhesives with different amounts of pTSA were cured in an electrically heated forced-draft oven at 150 °C. After curing, the cured adhesive was ground into powder (particle size 200 mesh). The powder was then wrapped in filter paper and immersed in water at a temperature of 63 ± 3 °C for 3 h. After immersion, the remaining solid was filtered and dried to constant weight. The water resistance residue rate of the cured adhesive was calculated by comparing the mass before and after immersion.

### 2.5. Fourier Transform Infrared Spectroscopy (FTIR) Test

The analysis was conducted using a Varian 1000 IR spectrometer (Varian, Palo Alto, CA, USA) in transmittance mode. The samples were scanned from 400 to 4000 cm^−1^ with a resolution of 4 cm^−1^.

### 2.6. Differential Scanning Calorimetry (DSC) Test

A differential scanning calorimeter (DSC 204 F1, Rodgau, Germany) produced by Netzsch was used for the test. The test parameters were as follows: temperature range 25–250 °C; heating rate 10 °C/min; nitrogen protection; and sample mass 5–8 mg.

### 2.7. Thermogravimetric (TG) Test

The cured adhesive powder was subjected to TG testing using a Netzsch TG 209 F3 thermogravimetric analyzer (Selb, Bavaria, Germany) under nitrogen protection, with a heating rate of 10 °C/min and a temperature range of 30–700 °C.

### 2.8. Scanning Electron Microscopy (SEM) Test

Adhesives with different amounts of pTSA were cured in an electrically heated forced-draft oven at 150 °C. After sputter coating the cross-section of the cured adhesive layer with gold, observations were made using a Zeiss GeminiSEM 300 scanning electron microscope from Tokyo, Japan.

### 2.9. Statistical Analysis

The data were processed using Excel 2021 and Origin 2024 software, and the significance of differences was judged via the one-way analysis of variance (ANOVA) (*p* < 0.05); error bars represent the standard deviation.

## 3. Results and Discussion

### 3.1. FT-IR Analysis

**Figure 1:**
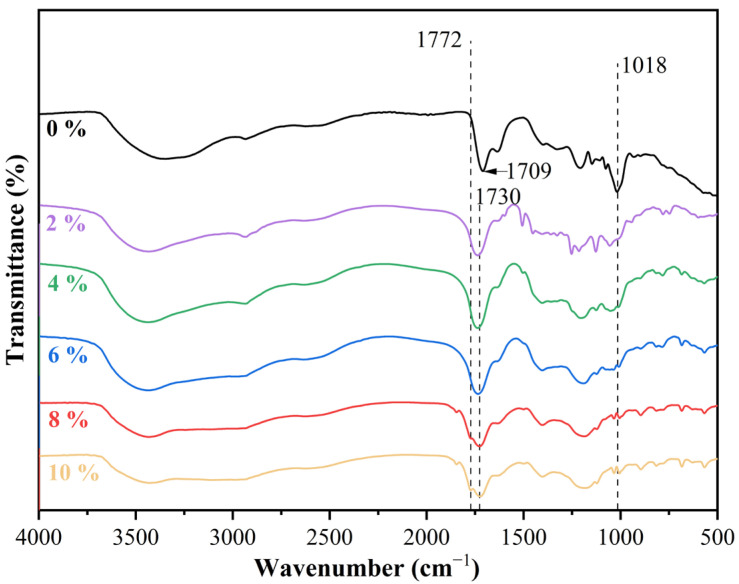
Effect of pTSA addition on the FT-IR spectra of starch–citric acid adhesive.

Figure 1 illustrates the effect of pTSA addition on the FT-IR spectra of starch–citric acid adhesive. The broad peak at 3500–3200 cm^−1^ is attributed to the stretching vibration of -OH, while the peak at 1018 cm^−1^ is associated with the bending vibration of -OH, both of which originate from the hydroxyl groups in starch and citric acid [30,31]. As the amount of the catalyst pTSA increases from 0% to 10%, the intensity of these peaks gradually decreases. This indicates that the hydroxyl groups are involved in the esterification reaction and are consumed, thereby reflecting a significant increase in the crosslinking degree of the adhesive with the increase in pTSA addition. The peak at 1709 cm^−1^ corresponds to the stretching vibration of the carboxyl C = O in citric acid. As can be seen from the figure, after the addition of pTSA, this peak undergoes a blue shift. This is because after the esterification reaction occurs, the C = O peak of the carboxylic acid shifts to 1730 cm^−1^ due to the disappearance of hydrogen bonds and the enhancement of the inductive effect, which is one of the important characteristics of the esterification reaction [32,33]. In addition, it is worth noting that, in the samples with 8% and 10% pTSA addition, a new absorption peak appears at 1772 cm^−1^, which is attributed to the ester carbonyl group, further confirming the formation of the ester group [34,35,36]. Furthermore, theoretically, pTSA also has the possibility of participating in esterification reactions. Overall, the addition of pTSA significantly promotes the esterification reaction of the adhesive, and with the increase in the addition amount, the reaction becomes more intense, thereby improving the crosslinking degree and properties of the adhesive.

### 3.2. Thermal Behavior Analysis

**Figure 2:**
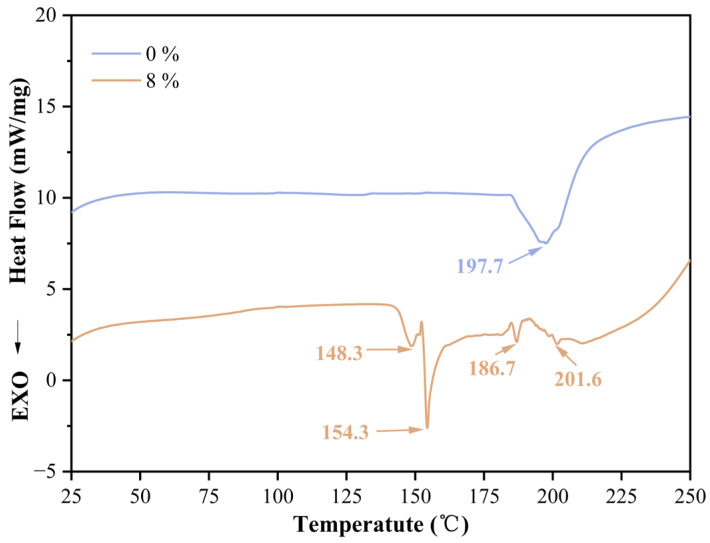
DSC curves of starch–citric acid adhesive with 0% and 8% pTSA addition.

As a precise thermal analysis technique, DSC plays a crucial role in studying the curing process of thermosetting resins and provides key information on the thermodynamic behavior of polymeric materials. A thorough understanding of the curing process of adhesives is vital for optimizing the hot-pressing process of plywood. As shown in Figure 2, the DSC curves clearly reveal the significant differences in thermal behavior between the sample without catalyst addition (0%) and the sample with 8% pTSA catalyst. After the addition of pTSA, the DSC curve shows multiple peaks at 148.3 °C, 154.3 °C, 186.7 °C, and 201.6 °C. During the heating process, the starch molecular chains undergo hydrolysis under the catalysis of pTSA, forming low-molecular-weight oligosaccharides and monosaccharides of varying lengths. These hydrolysis products may exhibit different thermal behaviors in DSC analysis, leading to the appearance of multiple peaks. In addition, the chemical reactions between pTSA and starch may produce a variety of intermediates and products. These intermediates and products have different chemical structures and thermal stabilities and are thus manifested as multiple peaks on the DSC curve. The main exothermic peak at 154.3 °C indicates that the addition of pTSA has significantly reduced the reaction peak temperature. This indicates that pTSA can significantly promote the esterification reaction between starch and citric acid, effectively reducing the activation energy of the reaction, thereby allowing the crosslinking reaction to proceed smoothly at a lower temperature. Moreover, the addition of the catalyst may also enhance the reaction efficiency, making the curing process of the adhesive system more rapid and complete, and thus significantly improving its thermal stability and final properties. This result fully confirms the excellent catalytic effect of pTSA in starch–citric acid adhesives, which not only effectively reduces the hot-pressing temperature but also shortens the reaction time, providing more energy-saving and efficient process conditions for plywood production.

**Figure 3:**
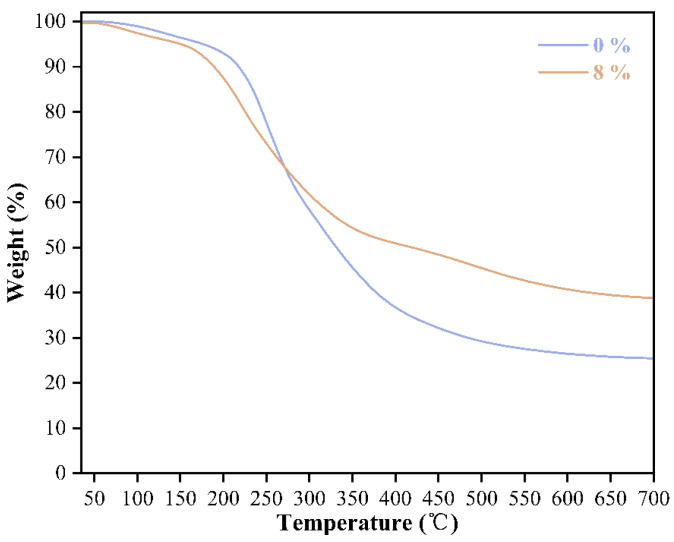
TG curves of starch–citric acid adhesive with 0% and 8% pTSA addition.

The influence of the introduction of the pTSA catalyst on the thermal stability and decomposition process of the starch–citric acid adhesive was analyzed by thermogravimetric (TG) and differential thermogravimetric (DTG) analysis of the adhesive samples. As shown in Figure 3, the curves of the two samples without catalyst addition (0%) and with 8% pTSA addition mainly show three stages. The first stage is the initial dehydration stage from room temperature to 150 °C. In this stage, both curves show a slight weight loss, which is mainly attributed to the evaporation of physically adsorbed water and a small amount of low-molecular-weight volatiles in the samples. The trajectories of the 0% and 8% samples in this stage are highly coincident, indicating that the addition of the catalyst did not significantly change the hygroscopicity of the system, and the loss of water mainly depends on the physical adsorption characteristics of the materials themselves. The second stage is the main thermal decomposition stage (150–500 °C). In this stage, the organic components of the adhesive underwent intense chemical decomposition, thus showing fundamental differences. The TG curve of the adhesive without pTSA addition dropped very steeply, indicating that the material underwent rapid and substantial decomposition within a narrow temperature range. This indicates that the uncatalyzed starch-citric acid mixture has an unstable structure, and once the thermal decomposition reaction is triggered, it proceeds rapidly with a large number of molecular chain breaks. The TG curve of the adhesive containing 8% pTSA has a much more gentle downward trend, which means that the weight loss rate of the 8% sample is slower throughout the heating process, directly proving its significant improvement in thermal stability. The third stage is the high-temperature residual carbon stage (500–700 °C). In this stage, the intense decomposition of organic matter has been basically completed, and the remaining material is the hard-to-decompose carbon residue. The adhesive with pTSA addition has a final residual carbon rate of 38.8%, significantly higher than that of the adhesive without pTSA addition (25.4%).

**Figure 4:**
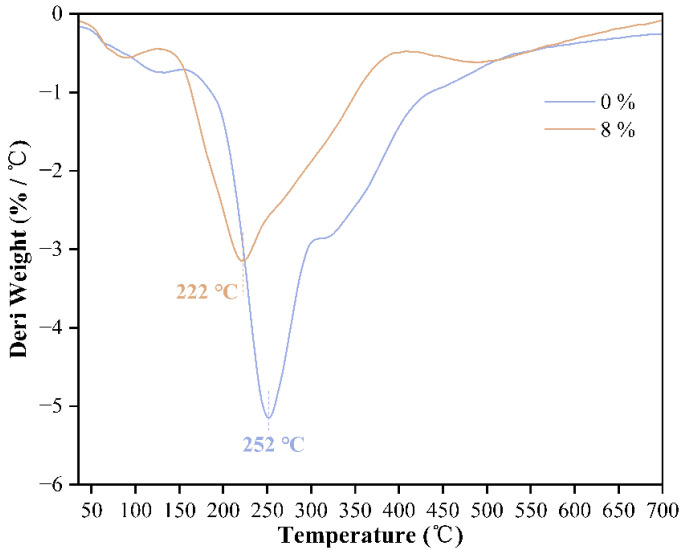
DTG curves of starch–citric acid adhesive with 0% and 8% pTSA addition.

In addition, the DTG curves in Figure 4 show that the DTG curve of the adhesive without pTSA addition has a strong and sharp main decomposition peak, indicating that its decomposition process is concentrated and intense with poor thermal stability. This is due to the low esterification degree and fragile molecular structure of the uncatalyzed starch–citric acid mixture, which quickly disintegrates when heated. The adhesive with pTSA addition shows a wider and more gentle decomposition peak, indicating that its decomposition process is more dispersed and its thermal stability is significantly improved. This further proves that the catalytic action of pTSA not only promotes the formation of the crosslinked network but also greatly improves its thermal stability and carbon-forming ability.

### 3.3. Bonding Performance Analysis

**Figure 5:**
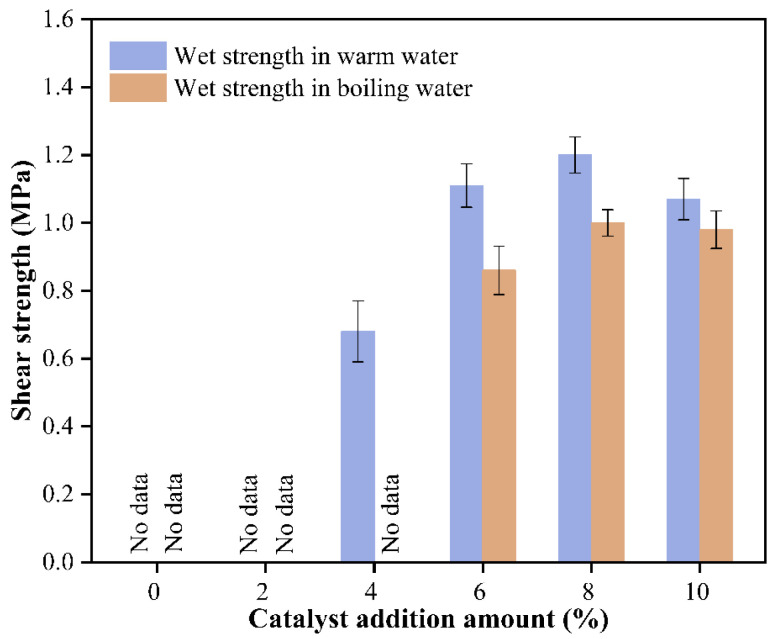
Effect of pTSA addition on the bonding performance of starch–citric acid adhesive.

As shown in Figure 5, the effect of pTSA as a catalyst on the bonding strength in the starch–citric acid wood adhesive system exhibits a clear pattern. Without the addition of a catalyst, plywood could not be successfully fabricated, even under hot-pressing conditions at 150 °C. When the catalyst concentration was 2%, the warm water strength and boiling water strength of the plywood were both zero, indicating that the adhesive had hardly formed an effective water-resistant bonding interface. The reason is that, at low catalyst concentrations, the esterification reaction between starch and citric acid is significantly limited. This reaction involves the formation of ester bonds between the hydroxyl groups of starch molecules and the carboxyl groups of citric acid. These bonds are essential for enhancing the adhesive’s water resistance and bonding strength. Insufficient pTSA could not effectively reduce the reaction activation energy and provide enough protons (H^+^) to catalyze the formation of ester bonds, resulting in the immediate disintegration of the adhesive layer upon contact with water.

When the catalyst concentration was increased to 4%, the warm water strength significantly increased to 0.68 MPa, which was a critical turning point, indicating that the esterification reaction had begun. However, the boiling water strength remained zero, suggesting that the crosslinked network formed at this stage was still very fragile and incomplete. In the mild warm water environment, some ester bonds and hydrogen bonds were retained, thus showing a certain degree of strength, but once exposed to the severe conditions of boiling water, these weak bonds were quickly destroyed, leading to the failure of the adhesive layer.

When the catalyst concentration reached 6%, the bonding performance made a qualitative leap. The warm water strength increased to 1.11 MPa, and the boiling water strength also reached 0.86 MPa, indicating the formation of a dense and complete three-dimensional network structure. An adequate amount of pTSA catalyst greatly promoted the esterification reaction, enabling starch molecular chains to achieve a high degree of crosslinking through citric acid ester bonds. This crosslinked network not only provided strong mechanical strength but, more importantly, endowed the adhesive layer with excellent water resistance, effectively resisting the erosion of boiling water. When the concentration was further increased to 8%, the performance remained at a very high level (warm water strength of 1.20 MPa and boiling water strength of 1.00 MPa). Within this range, the catalyst could fully meet the reaction requirements, and the esterification reaction tended to be complete, thus stabilizing the performance.

However, when the catalyst concentration reached 10%, both strength indicators showed a slight decrease (warm water strength of 1.07 MPa and boiling water strength of 0.98 MPa). This performance attenuation revealed the potential negative effects of excessive catalysis. The possible mechanisms for this phenomenon include the following: (1) Hydrolytic degradation of starch molecules: an excessively strong acidic environment (excess pTSA) may also catalyze the hydrolytic cleavage of glycosidic bonds in starch macromolecular chains, leading to a reduction in starch’s molecular weight, thereby weakening the cohesive strength of the adhesive; (2) over-crosslinking or increased brittleness: extreme catalytic conditions may cause local reactions to proceed too quickly, forming a highly tense or uneven crosslinked network, which instead makes the adhesive layer brittle and more prone to failure under actual shear stress; and (3) potential impact on wood interface: excess acid during hot pressing may cause slight hydrolysis of cellulose or hemicellulose on the wood surface, weakening the bonding strength between the wood itself and the adhesive layer at the interface.

Theoretically, pTSA can undergo an esterification reaction with starch. However, pTSA is too acidic and can promote the esterification reaction, but it is also prone to cause side reactions, leading to starch degradation and reducing the bonding strength. Moreover, curing of the adhesive requires crosslinking agents with a functional group degree greater than 2, but the functional group degree of pTSA is 1, which cannot meet the requirement. Therefore, although the esterification reaction between starch and pTSA is theoretically feasible, the bonding strength of the adhesive mainly comes from the esterification reaction between starch and citric acid. In summary, pTSA is an efficient catalyst for catalyzing the esterification reaction between starch and citric acid to prepare wood adhesives. In this experiment, there is an optimal range for the addition of pTSA, which is 6–8%. Within this range, the performance of the adhesive is the best. If the addition is too low, the esterification reaction will not be sufficient, resulting in suboptimal adhesive performance, whereas if the addition is too high, a series of side reactions may be triggered, leading to a decline in the adhesive’s performance.

### 3.4. Residue Rate Analysis

**Figure 6:**
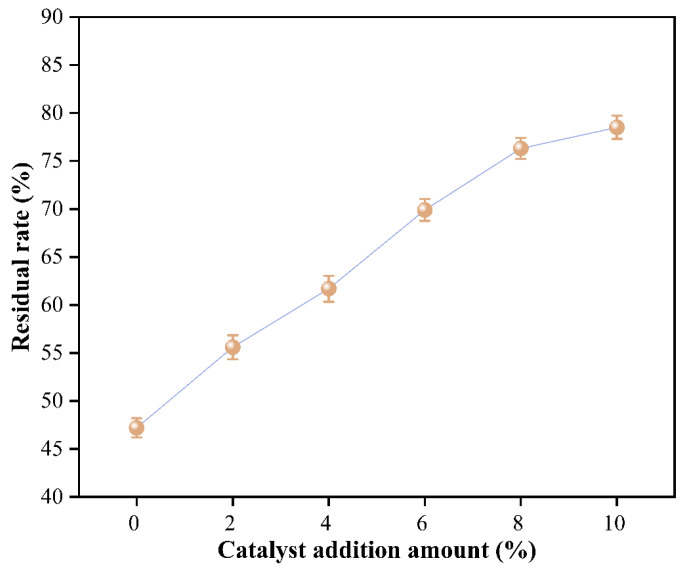
Effect of pTSA addition on the residue rate of cured products of starch–citric acid adhesive.

Water resistance is one of the key indicators for evaluating the performance of bio-based adhesives, and the residue rate is a more effective and direct method for measuring water resistance. As can be seen clearly from the trend in Figure 6, there is a significant positive correlation between the addition of pTSA and the residue rate of the adhesive. When the catalyst addition is 0%, the residue rate is at the lowest level, indicating that, in the uncatalyzed state, the crosslinking reaction degree of the starch–citric acid system is seriously insufficient, causing the adhesive layer to swell, hydrolyze, and even dissolve easily in hot water. As the catalyst addition increases gradually from 2% to 10%, the residue rate continues to rise. The chemical principle behind this phenomenon is very clear: pTSA, as a strong organic acid, can effectively provide a large number of protons (H^+^), thereby greatly promoting the esterification reaction between the hydroxyl groups (-OH) on the starch molecular chains and the carboxyl groups (-COOH) on citric acid molecules. A more efficient esterification reaction means that more and denser ester bond crosslinking points are formed in the three-dimensional network structure of the adhesive. When moisture tries to penetrate the adhesive layer, this dense crosslinking network can effectively resist the penetration and hydrolysis of moisture, thereby locking the polymer framework in place and showing a higher residue rate. This directly proves that the addition of the catalyst increases the crosslinking density, which is the fundamental reason for the improvement of water resistance.

**Figure 7:**
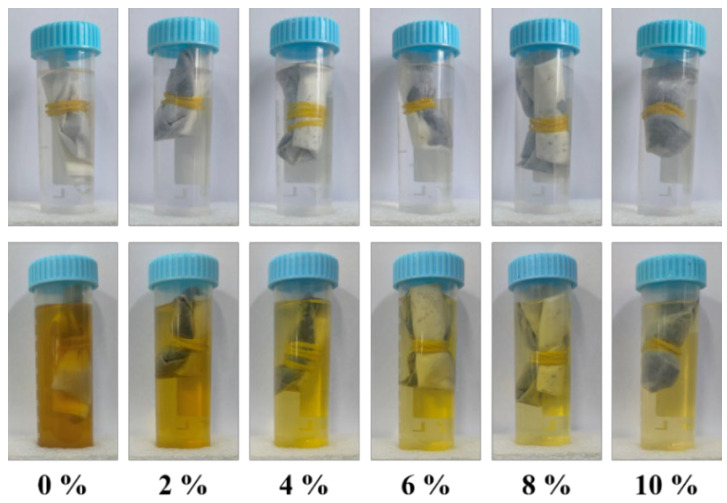
Photos of adhesive-cured products before and after soaking.

In addition, Figure 7 more intuitively shows the effect of pTSA addition on the water resistance of the adhesive. When the addition of pTSA is 0%, the solution after 3 h of hot water immersion appears turbid and has poor light transmittance, indicating that the adhesive has undergone significant swelling and partial dissolution in water. As the addition of pTSA increases, the light transmittance of the solution gradually increases, which further indicates that pTSA has a significant catalytic effect on starch–citric acid adhesives and can significantly improve the water resistance of the adhesive.

### 3.5. SEM Analysis

**Figure 8:**
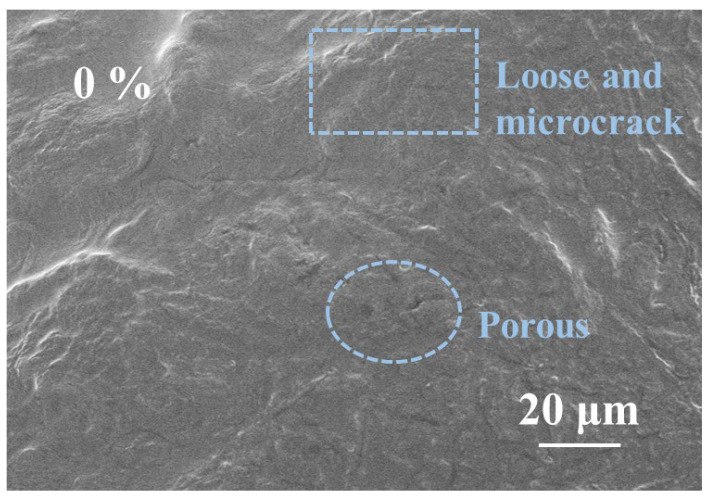
SEM image of starch–citric acid adhesive without catalyst.

Figure 8 shows the SEM images of the starch–citric acid adhesive without the addition of pTSA and with 8% pTSA addition. The cross-section of the cured product of the starch–citric acid adhesive prepared without a catalyst exhibits a distinctly loose and porous structure at the microscopic scale, accompanied by numerous microcracks. This structural characteristic indicates that, in the absence of a catalyst to promote the reaction, the crosslinking reaction within the adhesive system is not sufficient, the intermolecular forces are weak, and it is difficult to form a dense and stable three-dimensional network structure. Therefore, the overall polymer structure is relatively loose with poor coherence and contains many structural defects and weak areas. This microstructure is highly consistent with the thermal decomposition behavior shown in the thermogravimetric analysis, further confirming the instability of the material structure.

**Figure 9:**
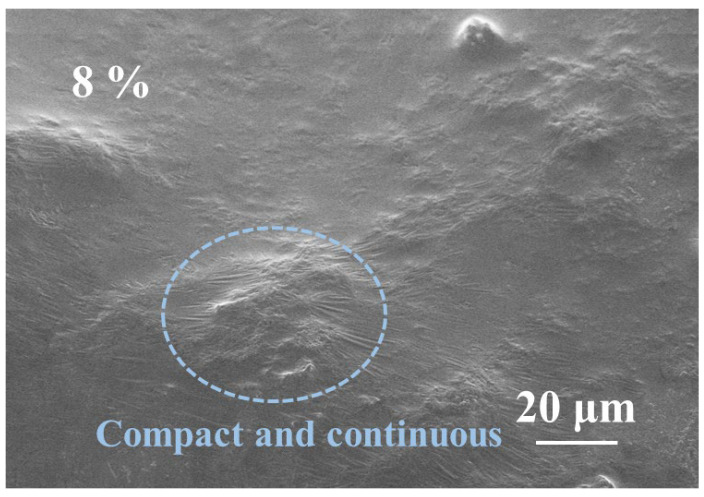
SEM image of starch–citric acid adhesive with 8% pTSA addition.

In contrast, when pTSA is introduced as a catalyst into the reaction system (Figure 9), the microstructure of the cross-section of the cured product undergoes significant optimization, presenting higher density and continuity, with no obvious pores or cracks. This transformation indicates that the addition of the catalyst significantly accelerates and optimizes the esterification reaction and crosslinking process between starch and citric acid, promoting the formation of a more complete and stable three-dimensional network structure. The molecular chains are interconnected through stronger covalent bonds and hydrogen bonds, making the microstructure of the entire adhesive system more uniform and robust. This structural enhancement is also verified in the thermogravimetric analysis, where the higher residual carbon rate indicates better structural retention of the material at high temperatures, proving a significant improvement in thermal stability.

Therefore, the introduction of pTSA not only improves the microstructural integrity of the adhesive but also significantly enhances the thermodynamic properties and mechanical strength of the final product, endowing it with better heat resistance and stress resistance capabilities in practical applications.

## 4. Conclusions

This study successfully utilized pTSA as an efficient catalyst to significantly promote the esterification crosslinking reaction between starch and citric acid, achieving a reduction in the hot-pressing temperature and an enhancement of the adhesive’s overall properties. (1) Significant catalytic effect: The addition of pTSA significantly reduced the activation energy of the esterification reaction, making it possible to lower the hot-pressing temperature. (2) Bonding performance improvement: When the addition of pTSA was 6–8%, the adhesive exhibited the best bonding strength (warm water strength of 1.11–1.20 MPa and boiling water strength of 0.86–1.00 MPa), with a significant improvement in water resistance. (3) Enhanced thermal stability: The thermal decomposition process of the adhesive catalyzed by pTSA was more gradual, and the residual carbon rate increased from 25.4% to 38.8%, indicating a significant enhancement in thermal stability and carbon-forming ability. (4) Improved microstructure: The cross-sectional structure of the adhesive catalyzed by pTSA was more dense and continuous, with no obvious pores or cracks, further confirming the perfection of the crosslinked network and the improvement of structural stability. (5) pTSA is an efficient and environmentally friendly catalyst suitable for the preparation of starch–citric acid adhesives. Within the addition range of 6–8%, it can significantly enhance the overall properties of the adhesive. (6) This adhesive, characterized by reduced hot-pressing temperatures and enhanced bonding properties, emerges as a sustainable and eco-friendly alternative to traditional formaldehyde-based adhesives. It is particularly well-suited for the wood panel industry and holds significant potential for other sectors such as furniture manufacturing, construction, and packaging, where both high performance and environmental sustainability are of paramount importance.

## Data Availability

The original contributions presented in this study are included in the article. Further inquiries can be directed to the corresponding author.
